# Obesity and Obesity Related Diseases, Sugar Consumption and Bad Oral Health: A Fatal Epidemic Mixtures: The Pediatric and Odontologist Point of View.

**Published:** 2017-07-01

**Authors:** Anna Pia Delli Bovi, Laura Di Michele, Giuliana Laino, Pietro Vajro

**Affiliations:** Pediatrics - Dept of Medicine, Surgery and Dentistry “Scuola Medica Salernitana”, University of Salerno -Via Allende, 84081 Baronissi, (SA) Italy

**Keywords:** added sugars, children, dental caries, obesity, oral health, sucrose, tooth decay

## Abstract

Obesity and dental caries are increasingly widespread pathologies. The former is growing so rapidly that the WHO classified its trend as an “epidemic”. Both are triggered by a number of well known common etiologic factors sharing also the high added sugar amount since childhood. Because of its fermentation and pH lowering, dietary sugar allows the cariogenic bacteria to damage the tooth enamel provoking the carious lesions.

WHO guidelines recommend reducing sugar intake to 10% of the total daily energy need, and highlight that there is evidence which suggests cuttingthis value down to 5% at least. The American guidelines addressing paediatric age put the limit to 25gr a day with a total ban on sugar in those aged 2 or less.

## I. THE PANDEMIC OF PEDIATRIC OBESITY

Obesity is a chronic disease characterized by a multifactorial aetiology with a complex interplay between genetic susceptibility and environmental factors. Since rates of overweight and obese adults and children in recent decades have dramatically increased worldwide, the World Health Organization (WHO) has classified this trend as a real obesity “epidemic” and the American Medical Association changed the term obesity from a *condition* into a *disease* in 2013 [1]**.** Obesity prevention in the paediatric age has been the object of several efforts at all levels but it has hardly obtained efficacious results [2]**.** Also the standard lifestyle changes-based treatment is flawed by high failure and/or relapse rates due to poor compliance at all ages. The unavailability of other ancillary pathomechanism-driven effective treatment strategies or better-tolerated treatments remains one of the main problems [1].

In recent years mounting evidence has suggested that

- sleep deprivation/fragmentation,
- diet rich in fructose,
- exposition to endocrine disruptors (e.g. Bisphenol A [BPA]), are emerging key factors most likely involved in the onset and treatment-resistance of obesity and obesity-related complications.

A pilot study focusing on these 3 factors in the Salerno’s area confirmed that they are additional suitable targets for future preventive and/or therapeutic approaches in paediatric obesity [1].

Also gut microbiota dysbiosis and altered intestinal permeability have recently been proposed to play a relevant role, and be a possible target for treatments of those cases unresponsive to diet and reduction of sedentary life [1; 3–6].

Here we will focus mainly on the pandemic sucrose consumption and obesity.

## II. THE PANDEMIC OF SUCROSE INTAKE

**Fig. 1:**
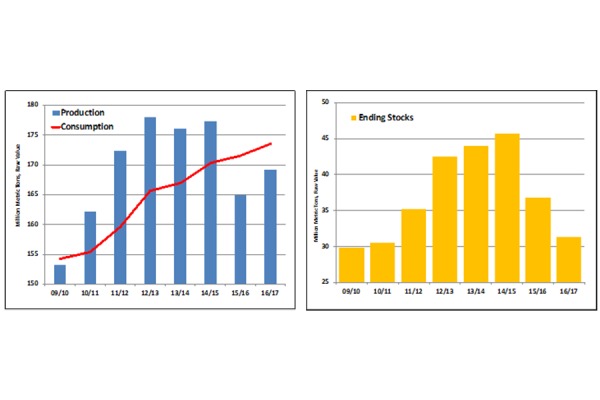
Global consumption (A) and drawing stocks (B)of sucrose for 2016/17

The pandemic of sucrose consumption appears endless. Global consumption for 2016/17 is forecast at a record 174 million metric tons (raw value), exceeding production (Fig. 1, **Left Panel** ) and drawing stocks down to the lowest level since 2010/11 (Fig. 1, **Right Panel**) [7].

Added sugars supply food energy but no other nutrients (empty calories) andincrease risk of developing obesity, cardiovascular disease, hypertension, obesity-related cancers, and dental caries[8].

Meta-analyses of randomized controlled trials (RCTs) in adults suggests an association between reduction of free sugars intake and reduced body weight in line with a comparable increase in body weight associated with an increased intake of free sugars [9]. Pediatric RCTs interventions comprising or including recommendations to reduce sugar-sweetened foods and beverages, instead were in general characterized by low compliance and no change in body weight. [9].

Fructose consumption is therefore a key contributor to the obesity epidemic, particularly in paediatric ages, as demonstrated by a series of studies that focused especially on sugar-sweetened beverages (e.g., soft drinks, commercial fruit juices, energy drinks, iced teas). This depends in particular on the lack of satiety after sweetened beverages which don’t influence food intake. Additionally it has been reported that fructose is associated with metabolic syndrome components including increased systolic blood pressure and insulin resistance. Results of our paediatric pilot study overall showed that grams of sugar per week and monthly and daily frequency of sugary drink consumption, reflect the excess body weight and metabolic syndrome clinical parameters, with the maximum intake per serving (>3 glasses per time) in 50% of the obese group. Furthermore, although the correlation between fructose consumption and blood pressure values was not statistically significant, the definitely hypertensive children (blood pressure ≥95th percentile) were among the main consumers of sweetened beverages [1].

## III. THE PANDEMIC OF DENTAL CARIES

Oral diseases are highly prevalent, by affecting approximately 3.9 billion people. WHO data show that untreated caries in permanent teeth is the most common condition evaluated for the entire GBD 2010 Study [10], with a global prevalence of 35% for all ages combined. The treatment of dental diseases absorbs from 5% to 10% of health budgets in wealthy countries, and obliges many people to travel to those countries where dentistry costs are lower [11–13].

**Fig. 2:**
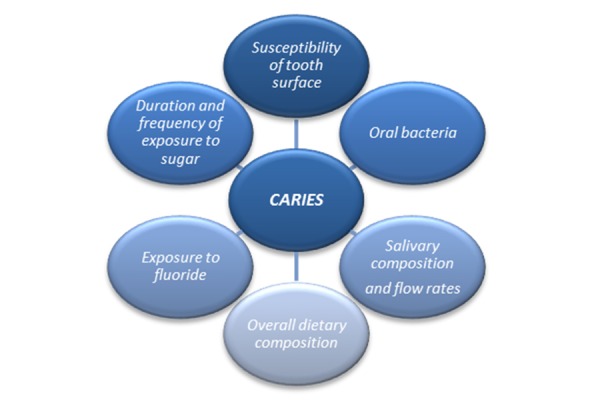
Dental caries: a multi-factorial disease

Dental caries is a multi-factorial disease, influenced by *individual* factors, as susceptibility of tooth surface and its structure, and *environmental* factors, as oral bacteria, exposure to fluoride, overall dietary composition, salivary composition and flow rates, duration and frequency of exposure to sugar (Fig. 2).

### Oral bacteria -

There are two specific groups of bacteria found in the mouth that are responsible for dental caries:

- Mutans streptococci (Streptococcus mutans), the main cause,
- Lactobacilli, associated with progression of the lesion.

They are found in large quantity in dental plaque. The tooth surface normally loses some tooth mineral with the action of the acid formed by bacterial plaque after ingestion of foods containing fermentable carbohydrates, which is the first step in the development of carious lesions.

Colonization by specific cariogenic bacteria is highly related to sucrose content of diet, and indeed in its absence these bacteria cannot colonize the mouth. Severe sucrose reduction in diet causes Mutans streptococci to reduce in number or disappear from the plaque, while frequent feeds of small quantities are more cariogenic [14].

### Fluoride -

Another important cariogenic factor is a limited exposure to fluoride. Fluoride protective role can be explained by the enhanced rate of remineralisation of enamel by saliva. In particular, fluorohydroxyapatite increases resistance of enamel to acid attack, although it does not completely prevent dental caries[9].

### Sugar -

An analysis of cohort studies in children suggests a positive association between the level of free sugars intake and dental caries. The evidence suggests higher rates of dental caries when the level is more than 10% of total energy intake compared with an intake < 10%. Furthermore, in three national population studies, less dental caries developed when per capita sugars intake was less than 10 kg/person/year (approximately 5% of total energy intake) [9].

## IV. ANTHROPOMETRIC MEASUREMENTS AND ORAL HEALTH: WHICH IS THE COMMON LINK ?

The association of anthropometric measurements and oral health is not surprising. In fact obese people have different composition of salivary bacteria and changes in the concentration of sialic acid, phosphorus and peroxidase activity, as well as reduced flow rate of stimulated saliva which may promote not only dental caries but also periodontal disease[15].

**Fig. 3:**
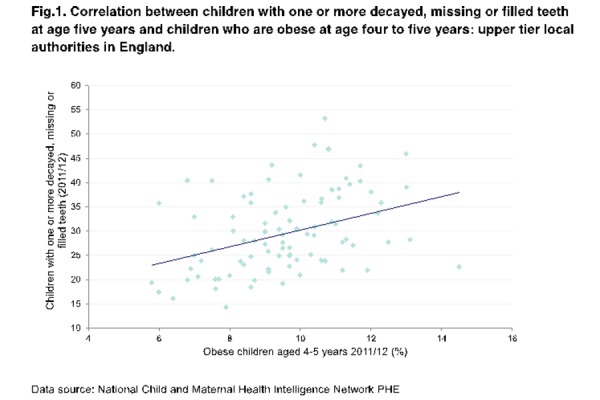
Correlation between obesity and caries prevalence (R=0.40) at age 5 years

Public Health outcome data routinely collected at age of five years old, provided an opportunity to examine if there is a relationship between dental caries and obesity prevalence in this age group at the local authority population level in England[16–17]. In a review of six studies with a positive association between obesity and dental caries in children and adolescents, the causal mechanism remains unclear. It is unknown if the reported associations occurred because there is a direct relationship between obesity and dental caries or if unhealthy diet, a factor common to both diseases, is responsible for this association. None of the studies in fact was designed to consider all the possible modifiers (e.g. access to health services, use of fluorides and oral health habits) and confounding factors (e.g. diet and socioeconomic status). These results were also comparable to those obtained by a systematic review and meta-analysis that included articles from the 2011–2012 literature [18–19]. Afterwards, another study showed that there is a weak to moderate correlation (R=0.40) between obesity and caries prevalence (Fig. 3), even if this observation is based only on ecological studies [17].

In another recent meta-analysis paediatric/adolescent obesity was significantly associated also with signs of periodontal disease including visible plaque index, bleeding on probing, subgingival calculus, probing depth and flow rate of salivary secretion [20].

## V. SUGAR: GENERAL RECOMMENDATIONS AND PRESENT GUIDELINES

Adult oral health is related to habits during childhood, which are in turn related to socioeconomic and psychosocial factors [21]. Indeed the chance of developing caries in permanent teeth is higher with greater DMFT-scores in primary teeth [22]. For this reason, the best strategy is the prevention in early age, mainly with a reduction of sugar intake which leads to a reduction of risk-factors.

### Less Sugar: how much less??

The majority of interviewed international cariology experts agrees that changes in sugar consumption policies have hitherto contributed considerably less to caries reduction as compared to the contribution of fluorides [23].

According to the WHO 2015 guidelines recommendations, the intake of free sugars could reduce the risk of developing dental diseases, with a particular focus on the prevention and control of unhealthy weight gain and dental caries. A reduced intake of added free sugars throughout the life course is recommended in both adults and children; the intake of free sugars should be reduced to less than 10% of total energy intake. A “conditional” reduction to below 5% of total energy intake would provide additional health benefits, but only a few epidemiological studies have been undertaken in population with a low sugar intake. In any case, three national population-wide studies compared the development of dental caries with sugars intakes of less than 5% of total energy intake *vs.* sugar intakes between 5–10% of total energy intake. These population-based ecological studies were performed during a period in which sugars availability dropped dramatically from 15 kg per person per year before the Second World War to 0.2 kg per person per year in 1946, anddemonstrated a reduction in dental caries [9].

According to Sheiham and James, in a paper published before the 2015 WHO guidelines, previous analyses, based on children, have in some way misled public health analyses on sugars. The recommendation that sugar intakes should be ≤10 % of energy intake is no longer acceptable. The high prevalence of dental caries in adult in fact underlines the need for very low sugar intakes throughout life, e.g. 2–3 % of energy intake, whether or not fluoride intake is correct [24].

Analogous recommendation has been issued by some relevant international medical societies which focused the pediatric age because the food a child eats in the first two years of age becomes, very easily, his favourite food. The American Heart Association for instance has now argued that the relation between added sugars and increased cardiovascular risk factor among US children are present at levels far below current consumption levels. Strong evidence supports the association of added sugars with increased cardiovascular disease risk in children through increased energy intake, increased adiposity and dyslipidemia. The committee found that it is reasonable to recommend that children consume ≤25 g (100 cal or ≈ 6 teaspoons) of added sugars per day and to avoid added sugars for children <2 years of age. Although added sugars can be safe if consumed in low quantity as part of a healthy diet, only few children respect such levels, making this an important public health target [8].

### Taxes on sugar-sweetened beverages [25] -

On 11 October 2016, in Geneva, the report titled “Fiscal policies for Diet and Prevention of Non Communicable Diseases (NCDs)” proposed to tax sugary drinks as a way to reduce obesity, type 2 diabetes and tooth decay as well. According to that document computations, fiscal policies that lead to at least a 20% increase in the retail price of sugary drinks would result in proportional reductions in consumption of such products [26].

In this regard, a recent experience in Mexico appears quite appealing. To prevent increase in obesity and diabetes, the Mexican government has in fact created a “sugar wall” by placing high taxes on non-essential sugar sweetened products. Authors of a study [27]report that soda sales in this country decreased for the first time as soon as 8% tax on soda and other junk foods was applied. The decline was particularly evident in households with low socioeconomic status. Overall, households cut their consumption of junk foods and sugar-sweetened beverages by 5%. These results remain extremely important because Hispanic children of Mexican origin have a high incidence of obesity and obesity-related nonalcoholic fatty liver disease (NAFLD). Susceptibility is probably linked to a combination of factors including the increasing epidemic of pediatric/adolescent obesity, a peculiar allele substitution in the PNPLA3 gene that reduces hepatic lipid catabolism, and an altered microbiome that may increase hepatic endotoxins. The combination of NAFLD and portal vein toxins secondary to intestinal dysbiosis appear to lead to the early occurrence of NAFLD progression to steatohepatitis (NASH), cirrhosis and early hepatocellular carcinoma [28].

## VI. FUTURE REVISION OF GUIDELINES

An update of the WHO guideline is planned for 2020. According to experts, updating of recommendations would much probably benefit from research in the following areas:

- How the intake of free sugars affects metabolism;
- Long term studies showing how changes in free sugars intake affect health;
- Thresholds above which the consumption of free sugars increases weight gain;
- The effectiveness of behavioural changes in reducing the intake of free sugars;
- Cohort studies to assess the risk of dental caries at different levels of sugar intake. [9]

## VII. OTHER IMPORTANT MEASURES

### More fluoride intake-

The finding during the first half of the 20th century of the link between natural fluoride, adjusted fluoride levels in drinking water and reduced dental caries prevalence has been a stimulus to assess the role of fluoride in improving oral health. Epidemiological studies of water fluoridation programmes have confirmed their safety and their effectiveness in controlling dental caries. Major advances in our knowledge of how fluoride impacts the caries process have led to the development, assessment of effectiveness, and promotion of other fluoride vehicles including salt, milk, tablets, toothpaste, gels. In 1993, the WHO convened an Expert Committee to provide continuing authoritative information on the role of fluorides in the promotion of oral health throughout the world [29–30] maybe capable of counteracting the alarming sugar/obesity epidemic.

### “Get enough sleep”-

Longitudinal analysis of a group of 6,316 children revealed that shorter sleep duration and higher salivary glucose levels independently from grade of obesity wereboth associated with increased gingival inflammation [31]. The measure adopted for the obesity fight might ultimately result useful also to oral health.

## VIII. CONCLUSION

Obesity and oral disease appear both linked to the intake of added sugar. According to 2015 WHO suggestions, it is important to reduce the sugar intake below the 10% of total calories for adults and children. It is necessary to pay attention especially to paediatric age, as adult oral health is rooted in early life conditions. In this phase of life decreasing of sugar intake to less than 25g would be advised, and children <2 years old should not assume added sugar at all. Increasing the total sleep hours would benefit obesity and dental caries as well.
